# Significant Molecular and Systemic Adaptations after Repeated Sprint Training in Hypoxia

**DOI:** 10.1371/journal.pone.0056522

**Published:** 2013-02-20

**Authors:** Raphael Faiss, Bertrand Léger, Jean-Marc Vesin, Pierre-Etienne Fournier, Yan Eggel, Olivier Dériaz, Grégoire P. Millet

**Affiliations:** 1 ISSUL-Department of Physiology, Faculty of Biology and Medicine, University of Lausanne, Switzerland; 2 Institute for research in rehabilitation, SuvaCare Rehabilitation Clinic, Sion, Switzerland; 3 Sport Medicine Unit, SuvaCare Rehabilitation Clinic, Sion, Switzerland; 4 Applied Signal Processing Group, Swiss Federal Institute of Technology, EPFL, Lausanne, Switzerland; University College London, United Kingdom

## Abstract

While intermittent hypoxic training (IHT) has been reported to evoke cellular responses via hypoxia inducible factors (HIFs) but without substantial performance benefits in endurance athletes, we hypothesized that repeated sprint training in hypoxia could enhance repeated sprint ability (RSA) performed in normoxia via improved glycolysis and O_2_ utilization. 40 trained subjects completed 8 cycling repeated sprint sessions in hypoxia (RSH, 3000 m) or normoxia (RSN, 485 m). Before (Pre-) and after (Post-) training, muscular levels of selected mRNAs were analyzed from resting muscle biopsies and RSA tested until exhaustion (10-s sprint, work-to-rest ratio 1∶2) with muscle perfusion assessed by near-infrared spectroscopy. From Pre- to Post-, the average power output of all sprints in RSA was increased (p<0.01) to the same extent (6% vs 7%, NS) in RSH and in RSN but the number of sprints to exhaustion was increased in RSH (9.4±4.8 vs. 13.0±6.2 sprints, p<0.01) but not in RSN (9.3±4.2 vs. 8.9±3.5). mRNA concentrations of HIF-1α (+55%), carbonic anhydrase III (+35%) and monocarboxylate transporter-4 (+20%) were augmented (p<0.05) whereas mitochondrial transcription factor A (−40%), peroxisome proliferator-activated receptor gamma coactivator 1α (−23%) and monocarboxylate transporter-1 (−36%) were decreased (p<0.01) in RSH only. Besides, the changes in total hemoglobin variations (Δ[tHb]) during sprints throughout RSA test increased to a greater extent (p<0.01) in RSH. Our findings show larger improvement in repeated sprint performance in RSH than in RSN with significant molecular adaptations and larger blood perfusion variations in active muscles.

## Introduction

Hypoxic conditions can be characterized in working muscle by a decreased oxygen tension (e.g. lower myoglobin oxygen saturation and intramyocellular oxygen partial pressure) [1]. There are some evidences that exercising in hypoxia affects muscular functions [2] and a large number of genes mediated by hypoxia-inducible factors (HIFs) [3]. These transcription factors have been demonstrated to control the expression of over 70 targets in response to a reduction in oxygen concentration. Intermittent hypoxic training (IHT) has been reported to evoke cellular responses via HIF1-α [4], [5] but without demonstrating substantial performance benefits in endurance athletes [6]–[8]. Actually, training intensity in hypoxia *per se* modulates molecular adaptations at muscular level with “adaptations that compensate for the reduced availability of oxygen during exercise” [9]. Then, any limitation in O_2_ availability (e.g. exercising in hypoxia) induces a compensatory vasodilation shifting blood flow upward to keep constant O_2_ delivery to the muscle. This later mechanism is also influenced by the exercise intensity [10]. For example, during supramaximal efforts, an increase in skeletal muscle blood flow allows matching the augmented O_2_ demand tightly [11].

Indeed, repeated sprints (RS) consist in maximal intensity exercise bouts with incomplete recoveries and could potentially challenge these adaptive mechanisms if performed in hypoxia. RS require huge energy amounts over short periods of time provided primarily by anaerobic glycolysis [12] with a progressive rise in the energy contribution of the aerobic metabolism [13]. One may therefore speculate that HIF-mediated molecular adaptations induced by repeated maximal efforts in hypoxia may challenge the functional reserve in muscle oxygen diffusing capacity likely utilized in hypoxia [14].

Besides, fast-twitch fibers (FT), preferentially recruited while sprinting [15], may better adjust to a high energetic demand with a greater fractional O_2_ extraction than their slow twitch counterparts [16] if oxygen tension falls in the muscle (e.g. in hypoxia). Consequently it can be hypothesized that RS training in hypoxia (RSH) could induce beneficial adaptations at the muscular level with an improved blood perfusion level inducing an enhanced O_2_ utilization by FT.

Nonetheless, to our knowledge, these mechanisms and the potential associated benefits for the ability to repeat sprints in normoxia have never been investigated in a randomized blind controlled study. The goal of our study was therefore to test putative additional benefit on systemic repeated sprint ability performance of RS training in hypoxia vs. normoxia and to assess blood perfusion and molecular responses at the muscular level.

## Methods

### Subjects

50 moderately trained male cyclists (age 35±7 years, height 179±5 cm, mass 75±9 kg) volunteered in the study and provided their written informed consent after the state Medical Ethics Committee approved the protocol (Commission Cantonale Valaisanne d’Ethique Médicale, CCVEM, Agreement 07/10, Sion, Switzerland). Subjects were all non-smokers lowlanders. None of the subjects were acclimatized or recently exposed to altitude and were asked to avoid any exposure to an altitude of more than 1500 m during the protocol.

### Study Design

Experimental protocol consisted in two testing sessions before (Pre-) and after (Post-) a four-week supervised specific training period (8 training sessions). Subjects were randomly assigned to one of the two treatment groups with repeated sprint training either in normoxia (RSN, n = 20) or hypoxia (RSH, n = 20). A control group (CON, n = 10) completed only Pre- and, after four weeks without specific training, Post-. Subjects were not specifically accustomed with 10 s cycling sprints but they were familiar with laboratory testing and longer high intensity cycling intervals. Protocol was run in single-blind fashion, as subjects both in RSH and RSN were told that all training sessions were held at altitude (without any accurate information on the altitude level). When asked, 95% of the subjects in RSH and 85% in RSN declared that their training was performed in hypoxia. Analyzes of blood samples and muscle biopsies were performed double-blinded.

### Supervised Training Protocol

Volunteers completed eight specific cycling training sessions with repeated sprints during four weeks starting the week after Pre- and finishing the week before Post-. Two sessions per week were completed in a hypoxic chamber (ATS Altitude, Sydney, Australia) built in our laboratory at an altitude of 485 m (Sion, Switzerland). The hypoxic chamber is a well-ventilated 30 cu. m room (2.4 m×5.0 m×2.5 m) with transparent glass panels. The system consists in a compressor storing air in pressurized tanks with serial connection to air filters allowing oxygen reduction (altitude simulation) in the air input flow to the chamber. For RSH, inspired oxygen fraction (F_i_O_2_) was set to 14.6% to simulate an altitude of 3000 m and controlled regularly with an electronic device (GOX 100 oximeter, Greisinger, Regenstauf, Germany). In order to blind subjects to altitude, the system was also run for RSN with a normoxic airflow into the chamber. Air input flow (up to 1000 l/min) was sufficient for safe, comfortable and stable training conditions. Temperature inside the chamber was maintained at 25° in average and three fans and crushed ice buckets were used to cool subjects.

### Training Sessions

**Figure 1:**
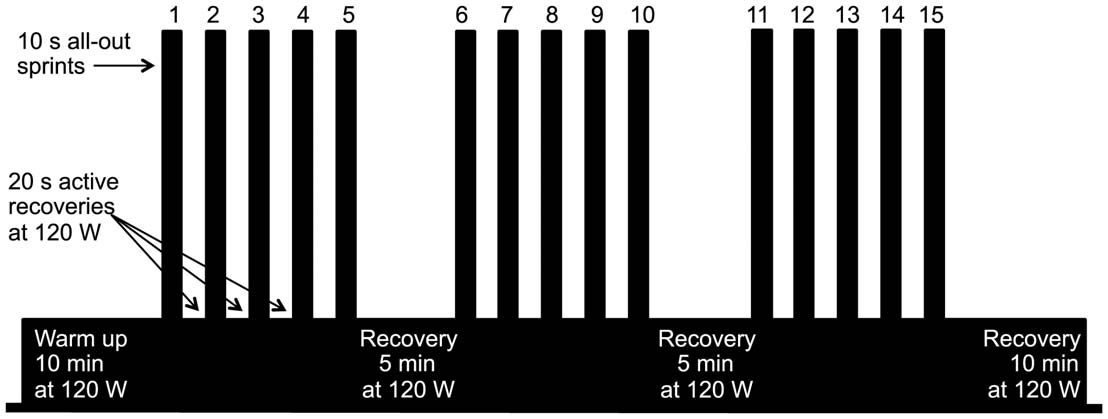
Description of a typical training session.

Following 10 min warm-up at 120 W, all training sessions consisted in 3 sets of 5×10 s all-out repeated sprints with a 5 min recovery period at 120 W between sprints and sets and ending with 10 min recovery at 120 W (Fig. 1). Subjects were instructed to perform all-out sprints trying to reach and maintain the highest power output for every sprint. Each session lasted 36 min 30 s and subjects spent ∼40 min in the chamber for every training session (320 min during the 4-weeks training period). Up to four subjects trained simultaneously in the chamber using their own road bicycle equipped with a power meter (Powertap Pro+ Wheel, Cycleops, Madison, USA) mounted on a stationary trainer with magnetic resistance (Powertap Supermagneto Pro, Cycleops, Madison, USA). Power output and heart rate were recorded for each training session. Electrolytes-carbohydrates sports drink (Isotonic, Sponser, Wollerau, Switzerland) and water were provided to ensure appropriate hydration and carbohydrate intake during training. After each session, subjects reported their rate of perceived exertion (RPE, Borg Scale, 6–20) and ticked the level of pain in the legs on a continuous visual analogic scale (VAS). Training sessions were scheduled with at least one day of rest in between for optimal recovery. Outside of the laboratory, volunteers were asked to maintain their usual endurance training avoiding any intense training and to fill a training diary.

### Baseline (Pre-) and Final (Post-) Tests

During the 48 h prior to each testing session, subjects were asked to refrain from any training or exhaustive activity. Due to the extreme intensity of the tests, subjects were asked not to report to the laboratory on an empty stomach. A standard breakfast (cornflakes or muesli with milk, bread with jam and water) was therefore advised. In all cases, subjects were asked to fill a nutritional diary for three days before each test and reproduce the last meals avoiding alcohol and caffeine intakes during the 24 h before each test. Tap water was provided *ad libitum* during the tests. Pre- and Post- trials were performed in the exact same sequence in normoxia in the well-ventilated laboratory at a constant temperature of 24°C.

### Blood Sampling and Muscle Biopsies

Upon arrival in the morning, subjects were weighed and a 2,5 ml blood sample was then taken from the antecubital vein for immediate hematocrit and hemoglobin concentration ([Hb]) evaluation. Gold standard hematocrit measurement was done by centrifugation of heparinized capillaries during 5 min at 13000 rpm (Haematokrit 210 Centrifuge, Hettich, Germany). [Hb] was assessed with a photometry device (Hemocue 201, Ängeholm, Sweden). Blood lactate concentration ([La]) was measured from a capillary fingertip sample (Lactate Pro, Arkray, Japan) at rest and immediately after each test.

Resting skeletal muscle microbiopsy samples were taken under local anaesthesia (Rapidocaine®, 1% plain) of the left leg from the belly of the *vastus lateralis* muscle using percutaneous needle biopsy technique (Pro-Mag, Medical Device Technologies Inc., Gainsville, USA) [17]. Three individual muscle samples (between 60 and 90 mg of muscle) were taken within less than 1 min from one single incision. The muscle samples were washed in saline solution then immediately frozen in liquid nitrogen for RNA and protein extraction. For practical reasons muscle biopsies were taken in 5 subjects only in CON.

### RNA Extraction and Real Time Quantitative PCR

RNA from skeletal muscle (approximately 25 mg of muscle) was extracted using a commercially available preparation, peqGOLD Tri-Fast (Peqlab, Germany). Five micrograms of RNA were reverse transcribed to cDNA using Random Hexamer primers (Promega AG, Switzerland) and AffinityScript Multiple Temperature Reverse Transcriptase (Agilent Technologies Inc, Santa Clara, CA, USA), while quantitative PCR was performed using an MX3000p thermal cycler system and Brilliant SYBER Green QPCR Master Mix (Agilent Technologies). PCR primers sequences are listed in Table 1 and PCR conditions were used as published previously by our research group [17], [18]. To control for any variations due to efficiencies of the reverse transcription and PCR, acidic ribosomal phosphoprotein P0 (RPLP0 or 36B4) was used as an internal control. All PCR runs were performed in triplicate.

**Table 1:** Quantitative PCR primer sequences.

Gene	Sequence 5–3	Temperature
MCT-1	Sense CCA AGG CAG GGA AAG ATA AGT CT	60
	Anti ATC TTT TTT CAC ACC AGA TTT TCC A	
MCT-4	Sense GCA CCC ACA AGT TCT CCA GT	60
	Anti CAA AAT CAG GGA GGA GGT GA	
CA3	Sense GTC CTC TCC CTG GAC CCT AC	60
	Anti TTG TCC AAT GCA TCA AGG AA	
Tfam	Sense CCA AAA AGA CCT CGT TCA GCT TA	60
	Anti TGC GGT GAA TCA CCC TTA GC	
Mb	Sense GCA TGC CAC CAA GCA CAA G	60
	Anti TGA TGC ATT CCG AGA TGA ACT C	
PGC-1a	Sense GGT CTC TCC TTG CAG CAC AAG	60
	Anti CTG GGA TGA CCG AAG TGC TT	
VEGF	Sense CCT TGC TGC TCT ACC TCC AC	60
	Anti ATC TGC ATG GTG ATG TTG GA	
HIF-1a	Sense TCC ATG TGA CCA TGA GGA AA	60
	Anti CCA AGC AGG TCA TAG GTG GT	
RPLP0	Sense GTG ATG TGC AGC TGA TCA AGA CT	60
	Anti GAT GAC CAG CCC AAA GGA GA	

MCT-1, monocarboxylate transporter 1; MCT-4, monocarboxylate transporter 4; CA3, carbonic anhydrase III; Tfam, transcription factor A mitochondrial; Mb, myoglobin; PGC-1α, peroxisome proliferator-activated receptor gamma, coactivator 1 alpha; VEGF, vascular endothelial growth factor A; HIF-1, hypoxia inducible factor 1, alpha subunit; RPLP0, ribosomal protein large P0.

### Enzyme Activities

20 mg of tissue were used to measure enzyme activities of interest in RSH and RSN but not in CON. Lactate dehydrogenase (LDH) activity was determined using a colorimetric method developed by BioAssay system (QuantiChrom) according to manufacturer’s instructions. The non-radioactive colorimetric LDH assay is based on the reduction of the tetrazolium salt MTT in a NADH-coupled enzymatic reaction to a reduced form of MTT which exhibits an absorption maximum at 565 nm [19]. Measurement of citrate synthase (CS) activity was performed by linking the release of Coenzyme-A to the colometric agent DTNB 5,5-dithiobis-2-nitrobenzoate. Changes in absorbance were followed at 412 nm [20].

### Exercise Performance Tests and Measurements

An electronically braked cycling ergometer (Lode Excalibur Sport, Groninge, The Netherlands) equipped with clip-on pedals was used for all performance tests. Seating and handlebar positions were adjusted individually for all subjects and reproduced for all tests. For each performance test, maximal instantaneous power output and pedaling frequency (PF) were recorded and average power output calculated. All tests were performed against an individual fixed torque of 0.8 Nm^.^kg^-1^ with the ergometer in manufacturer’s “Wingate mode” unless otherwise specified. RPE and leg pain were assessed as described above.

### Near-infrared Spectroscopy Measurements

**Figure 2:**
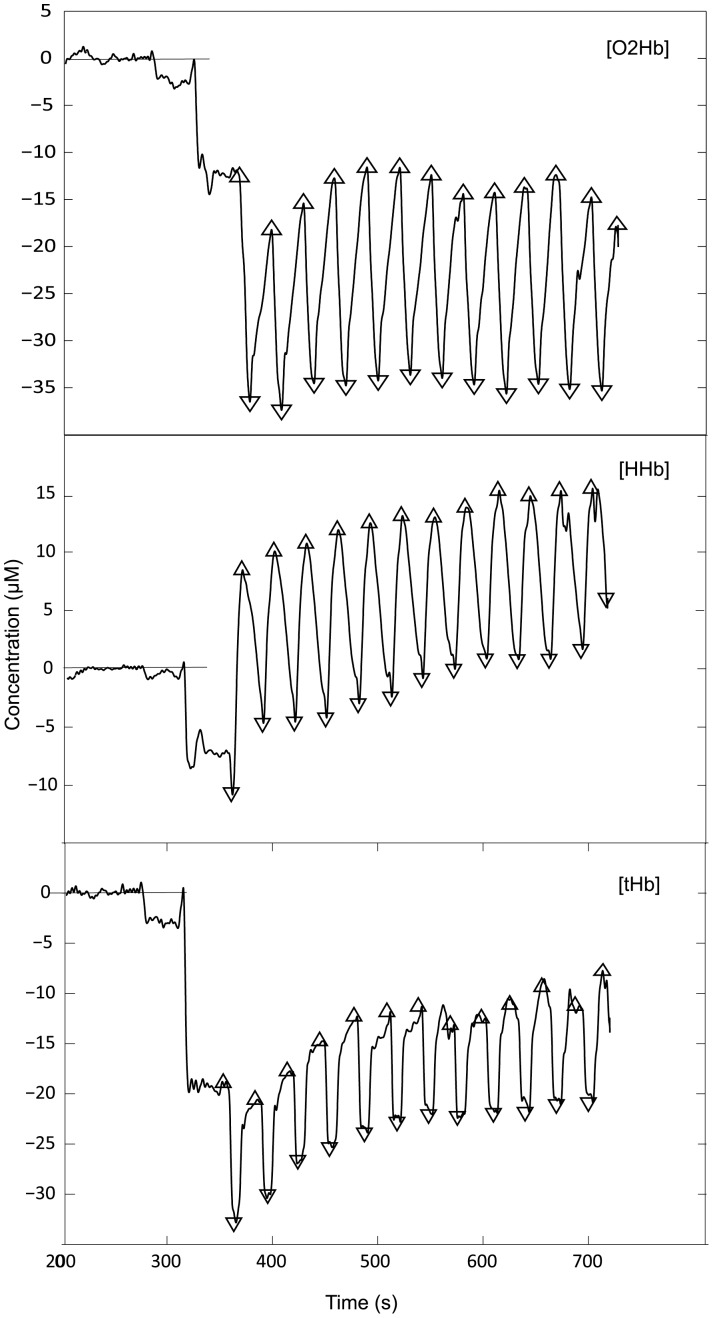
Typical signals recorded by near-infrared spectroscopy during repeated sprint ability test after signal treatment, maximum and minimum determination and smoothing. Concentrations of O_2_Hb, HHb and tHb are expressed as changes in µM from resting baseline set to 0 µM.

Muscle oxygenation was assessed using a near-infrared spectroscopy (NIRS) technique well described elsewhere [21]. The NIRS device (Oxymon Mk III, Artinis, Zetten, The Netherlands intramuscular oxygenation) was used to measure changes in muscle oxygenation by placing a double optode sensor on *m*. *vastus lateralis* on the right leg, at mid thigh with an interoptode spacing of 40 mm. The probe was attached to the skin with double-sided tape and firmly fastened with an opaque cotton elastic band wrapped around subjects’ thigh. Position of the probe was marked during Pre- with a permanent pen for accurate repositioning in the Post-trial. A standard differential pathlength factor (DPF) of 4.0 was used in lack of any clear standard value for human quadriceps muscle during cycling sprints [22], [23]. All signals were recorded with a sampling frequency of 50 Hz. They were down sampled at 10 Hz using Matlab (Matlab Software, Nattick (MA) USA) routine resample. Then a 10th-order low-pass zero-phase Butterworth filter (cutoff frequency 0.1 Hz) was applied to the resampled signals in order to remove possible artifacts and smooth the pedaling-induced perturbations. Automatic detection of the starting and end times of the successive sprints was obtained by estimating the filtered desoxyhemoglobin signal upper and lower envelopes using local minima and maxima in a sliding window of length 400 samples (4 s). The starting and end times were obtained as the times of contact between the envelopes and the signal. This allowed the determination of maximum and minimum for each signal during the successive sprint and recovery phases. Concentrations for oxyhemoglobin ([O_2_Hb]), desoxyhemoglobin ([HHb]) and total hemoglobin/myoglobin ([tHb]) were determined. Figure 2 illustrates the typical signals recorded by NIRS. Since [HHb] values were proposed to be less sensitive than [O_2_Hb] to blood flow variations [24] and changes in O_2_Hb signals might be confounded by rapid blood volume changes during sprints [25] only [HHb] and [tHb] were analyzed for relevant interpretations. Differences between maximum and minimum concentrations were defined as the amplitude of the variation for each sprint (Δ[tHb] and Δ[HHb]). Thus, for example, at the beginning of each sprint a minimum value for [HHb] is measured whereas [HHb] reaches a maximum at the end of each sprint. Conversely, a maximum in [tHb] is observed at the beginning of each sprint (i.e. end of each recovery period) and [tHb] decreases to reach a minimum value at the end of each sprint. The amplitudes of the first sprint were standardized as 100%. Amplitudes during the following sprints and the average value for all sprints throughout the RSA test (i.e. Δ[HHb]_av_ and Δ[tHb]_av_) were calculated. The same analysis was performed during the successive recovery phases. In addition, the Δ[tHb]/Power was calculated as the ratio between Δ[tHb] and mean power for each sprint as an index of blood perfusion relative to the intensity of each sprint.

### Electromyography Acquisition and Analysis

Surface EMG of the vastus lateralis (VL) and biceps femoris (BF) muscles on the right leg were recorded during the tests using MP36 hardware (Biopac Systems Inc., Santa Barbara, CA) with specific electrodes (EL-503, Biopac) with a diameter of 35 mm pasted longitudinally on the distal part of the muscle belly with an center-to-center distance of 25 mm. A ground electrode was located on the patella. Prior to electrode placement, skin on the surface of these muscles was carefully shaved, abraded and cleaned (impedance <5 kΩ). EMG cables running inside subjects’ cycling tights prevented cables movement. The myoelectric signal was collected at 2000 Hz, amplified and filtered (pass band 5–500 Hz, gain = 1000). EMG parameters were then computed in a dedicated software (AcqKnowledge 4.1.0, Biopac Systems Inc., Santa Barbara, CA). During RS, each muscle activity was determined by calculating the mean value of the root mean square (RMS) of the signal for each sprint. Average values were then calculated for the entire duration (10 s) of each sprint.

### Repeated Sprint Ability Test

Approximately 20 min after the resting biopsy, subjects performed a 5 min warm-up at 120 W at their preferred PF followed by an isolated 10 s sprint, 4 min 50 s recovery at 120 W and a second isolated 10 s sprint. 15 s after this second sprint, subjects were asked to stop pedaling and rest keeping the right leg motionless and straight during 5 min for NIRS baseline measurement. Subjects then started pedaling for 45 s against a 20 W resistance at a PF of approximately 85 rpm followed immediately by the repeated sprint ability (RSA) test with a 1∶2 work-to-rest ratio (10 s all-out sprint and 20 s rest). Immediately after each sprint, the ergometer switched automatically to a resistance of 20 W during the 20 s active recovery phase. Subjects were given very strong verbal encouragement and performed as many sprints as possible until exhaustion (i.e. task failure). To avoid any protective pacing strategy, average power during the first two sprints was controlled to reach at least 95% of the best average of the two isolated sprints, which was the case in all subjects. Subjects were never given any indication on the number of sprints performed. A minimal PF of 70 rpm after 5 s or less of sprinting was set as a criterion to stop subjects’ test. However, volitional exhaustion was observed in all subjects before this criterion was reached. Unfinished sprints (subjects not being able to turn pedals anymore) where not taken into consideration.

### 3 min All-Out Test

45 min after the RSA test, subjects performed a 3-min all-out test following a protocol well described elsewhere [26] in order to determine 3-min average power output. Strong verbal encouragement was given throughout the test and PF was instructed to be as high as possible all time to elicit maximal all-out performance. Information about elapsed time was only given during the final minute every 15 seconds in order to prevent pacing and further motivate subjects. A peak in oxygen consumption (VO_2peak_) was calculated as the highest 30 s average value during the test with a gas exchange analyzer (Metalyzer, Cortex Medical, Leipzig, Germany). Although residual fatigue from the RSA test was probable, this test was performed for all subjects at the same moment of the testing sequence allowing for a good comparison of subjects’ aerobic capacity (average power during 3 min). For this test, the ergometer was set in linear mode (power output increases as PF increases).

### 30 s Wingate Test

Prior to the first and last training sessions, subjects performed a standard 30 s Wingate test [27] after a 6 min warm-up at 120 W and the average power during the 30 s was recorded.

### Statistical Analyses

Data are presented as mean (SD). Performance and blood perfusion changes during RSA test were first evaluated with a 2-way (training group×sprint number) general linear model repeated-measures ANOVA with all pairwise multiple comparison procedures (Holm-Sidak method). Performance improvement, muscle oxygenation during RSA, mRNA concentrations, blood lactate and other single variables test were then evaluated with 2-way (training group×time (Pre- vs. Post-)) general linear model repeated-measures ANOVAs with all pairwise multiple comparison procedures (Holm-Sidak method). All analyses were made using Sigmaplot 11.0 software (Systat Software, CA, USA). Null hypothesis was rejected at p<0.05.

## Results

Total work and training intensity during supervised RS training were similar during RSN and RSH, with only the mean heart rate being higher in RSH (Table 2). In addition to specific training in the laboratory, subjects trained similarly for 5.0±2.5 h and 5.1±2.2 h for the RSH and RSN groups, respectively.

**Table 2:** Total work and measured parameters during all training sessions in the hypoxic (RSH) and normoxic (RSN) training groups.

	RSH	RSN
Total work (kJ)	2728±242	2738±335
Mean peak power output (W)	960±117	1003±146
Mean heart rate (bpm)	151±12	141±12#
Mean RPE	16.2±1.4	16.7±1.5
« Pain in the legs » VAS	7.0±1.9	7.6±0.9

RPE, Rate of perceived exertion (Borg 6–20 scale); VAS, Visual analogic 1–10 scale.

# p<0.05 for difference with RSH.

### Performance

**Figure 3:**
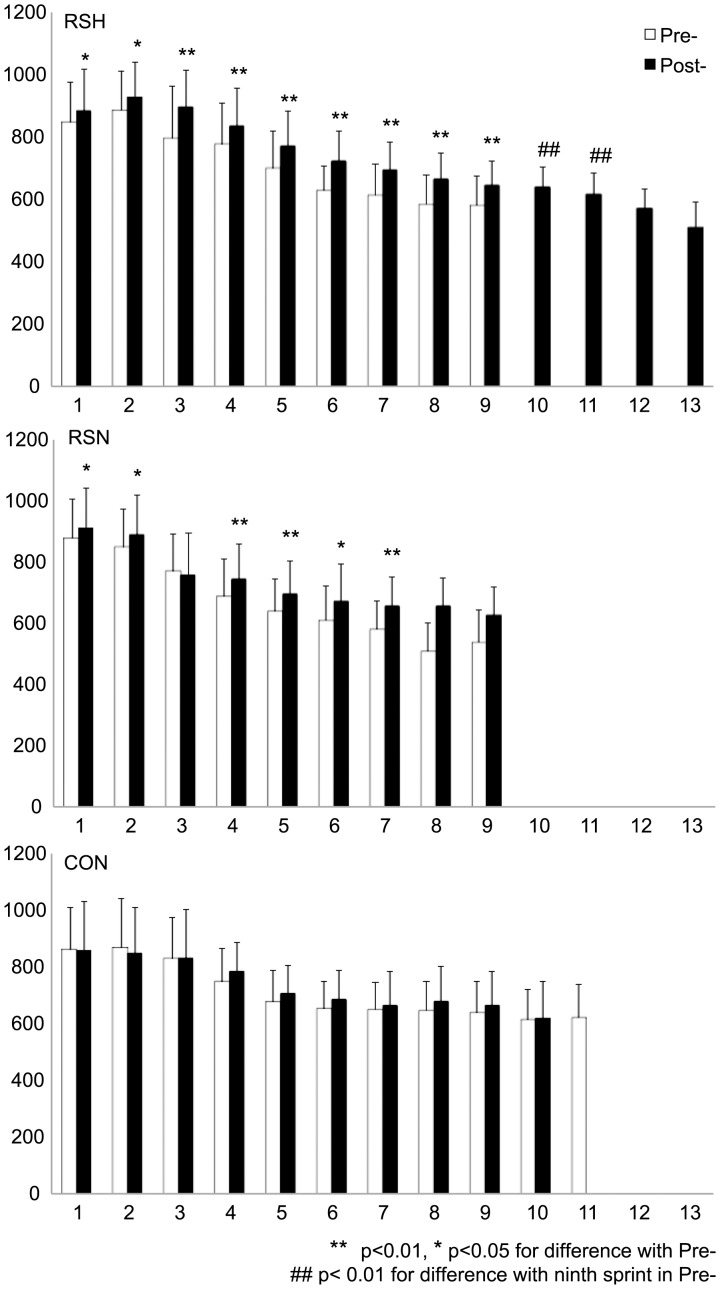
Average power output (W) in successive sprints during the repeated sprint test before (Pre-) and after (Post-) the specific repeated sprint training in hypoxia (RSH), in normoxia (RSN) or in control group (CON).

From Pre- to Post-, the average power of all sprints during the RSA test increased (p<0.01) to the same extent (6±7% vs. 7±8%, NS) in RSH and in RSN, respectively, but not in CON (2±5%, NS). The number of sprints prior to exhaustion was increased in RSH (9.4±4.8 vs. 13.0±6.2, p<0.01) but not in RSN (9.3±4.2 vs. 8.9±3.5, NS) nor in CON (11.0±7.1 vs. 10.3±6.2, NS). In Post- compared to Pre-, 10-s average power in the successive sprints was significantly improved till the ninth sprint in RSH and till the seventh in RSN. In RSH, this 10-s power output was significantly better (p<0.01) in the 10th and 11th sprints in Post- than the ninth sprint in Pre- (Fig. 3). Significant group (RSH vs. RSN) by time (Pre- vs. Post-) interactions were found in the number of sprints (F = 24.22; p<0.001) and total work (F = 27.07; p<0.001) performed during the RSA test. Subjects reached volitional exhaustion at the same relative power output (e.g. % of the best sprint) in RSH (67±11% vs. 69±8% for Pre- vs. Post-, respectively, NS) and in RSN (65±7% vs. 68±9%, NS). From Pre- to Post-, average PF during the last 5 s of all successive sprints was improved to the same extent (p<0.01) in RSH (96±14 vs. 104±11) and in RSN (100±11 vs. 110±10) but not in CON (103±2 vs. 103±3, NS). The maximal PF of each sprint was higher (p<0.01) in Post- both in RSH (124±11 vs. 130±8) and in RSN (125±8 vs. 131±8) but not in CON (129±3 vs. 130±4, NS).

The average power during the 3-min all-out test as well as hematocrit and hemoglobin concentration at rest were unchanged between Pre- and Post- in all groups (Tables 3 and 4). RSH and RSN improved single 10-s sprint and Wingate performances (p<0.01) similarly whereas it did not change in CON (Table 4).

**Table 3:** Subjects’ characteristics before (Pre-) and after (Post-) repeated sprint training in hypoxia (RSH), in normoxia (RSN) or in the control group (CON).

	RSN		RSH		CON	
	Pre-	Post-	Pre-	Post-	Pre-	Post-
Body mass (kg)	73.7±7.7	73.7±8.1	76.8±10.4	76.5±10.1	76.4±11.0	75.8±11.3
Hematocrit (%)	45.6±2.7	45.4±2.2	44.2±2.7	44.8±2.4	44.9±2.6	45.7±2.7
Hemoglobin(g^.^l^−1^)	152±12	154±12	153±12	154±16	154±8	157±9
VO_2peak_(l^.^min^−1^)	3.8±0.5	4.0±0.4	3.8±0.4	3.8±0.4	3.9±0.5	3.9±0.5

VO_2peak_, peak in oxygen consumption measured during 3-min all-out test.

**Table 4:** Performance results before (Pre-) and after (Post-) repeated sprint training in hypoxia (RSH), in normoxia (RSN) or in the control group (CON).

	RSH		RSN		CON	
	Pre-	Post-	Pre-	Post-	Pre-	Post-
Single 10-s sprint average power (W)	870±132	925±120**	879±131	940±131**	890±151	877±163
RSA Peak Power (W)	1365±311	1500±217**	1351±226	1427±275*	1357±275	1377±322
RSA Peak heart rate (bpm)	173±14	175±11	172±8	172±8	171±10	171±11
RSA Best average power (W)	870±133	925±120**	879±131	940±131**	870±151	877±163
RSA Mean power of allsprints (W)	699±97	737±84**	693±120	734±104**	726±115	747±125
RSA [La] (mmol^.^l^-1^)	15.0±2.3	15.4±2.1	14.2±1.7	14.8±1.6	14.8±2.0	13.8±1.5
RSA Average RPE	17.3±1.5	17.4±1.7	17.8±1.4	17.8±1.5	17.8±1.0	17.6±0.8
RSA Average « Pain in thelegs » VAS	5.2±2	6.7±1.9**	5.8±1.9	7.7±1.7**	6.4±2.3	6.1±1.8
30 s Wingate averagepower (W)	699±102	718±94**	688±75	723±86**	670±86	689±105
30 s Wingate [La] (mmol^.^l^−1^)	11.0±2.2	12.0±2.0	11.3±2.3	11.0±2.2	10.8±3.0	11.4±2.5
30 s Wingate Average RPE	15.3±2.0	16.1±1.9	16.3±1.6	17.0±1.6	15.8±1.7	16.0±0.9
3-min all-out averagepower (W)	368±45	383±39	371±49	382±47	385±48	378±48
3-min all-out [La] (mmol^.^l^−1^)	14.7±2.1	15.0±1.9	14.5±2.0	15.1±2.0	14.2±2.6	13.8±2.4
3-min all-out RPE	17.5±1.7	18.0±1.7	18.1±1.8	18.7±1.1	17.8±1.0	17.9±1.0

RSA, repeated sprint ability test; [La] Blood lactate concentration after repeated sprint test; RPE, rate of perceived exertion (Borg 6–20 scale); VAS, visual analogic scale (1–10) score.

** p<0.01,

* p<0.05 for difference with Pre-.

### Muscle Oxygenation

#### Repeated sprints

**Figure 4:**
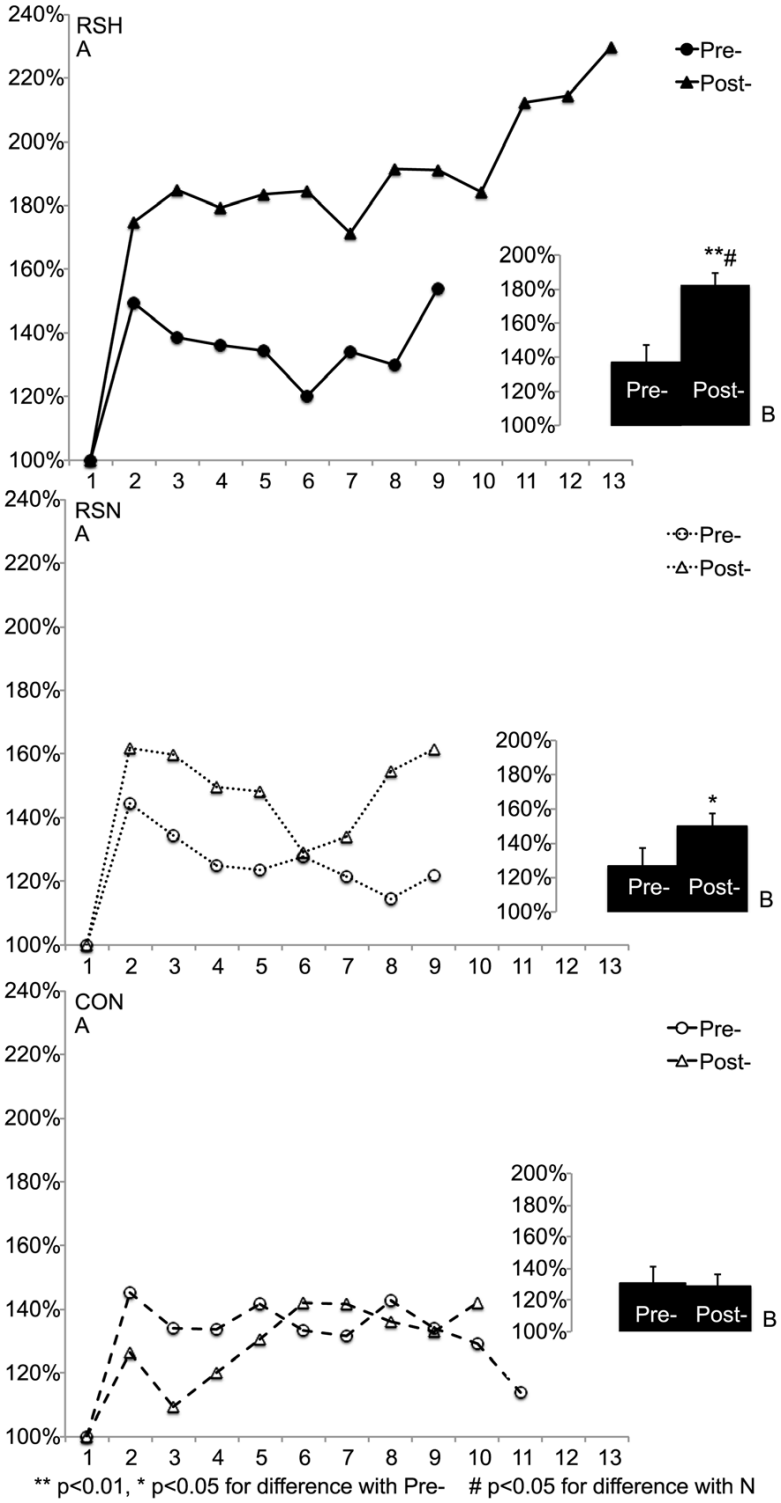
Δ[tHb]: successive changes in total hemoglobin concentrations’ amplitude during sprints (expressed in percent compared to the first sprint) measured by near infrared-spectroscopy (A) and Δ[tHb]_av_: average of all changes during the repeated sprint test to exhaustion (B) before (Pre-) and after (Post-) the specific repeated sprint training in hypoxia (RSH), in normoxia (RSN) or in control group (CON).

After training, Δ[tHb]_av_ increased to a greater extent (F = 15.8, p<0.01) in RSH than in RSN and was not different in Pre- and in Post- in CON (Fig. 4B).

Meanwhile, Δ[HHb]_av_ increased similarly in RSH (from 79±6% to 98±5%, p<0.01) and in RSN (from 106±3% to 120±10%, p<0.01) but was not different in Pre- and in Post- in CON (118±9% vs. 121±13%, NS).

The average Δ[tHb]/Power ratio increased to a greater extent (F = 9.1, p<0.05) in RSH (from 1.64±0.25 to 2.23±0.35, p<0.01) than in RSN (from 1.68±0.15 to 1.98±0.30, p<0.05) and was not different Pre- compared to Post- in CON (1.61±0.26 vs. 1.56±0.33, NS).

#### Recovery phases

**Figure 5:**
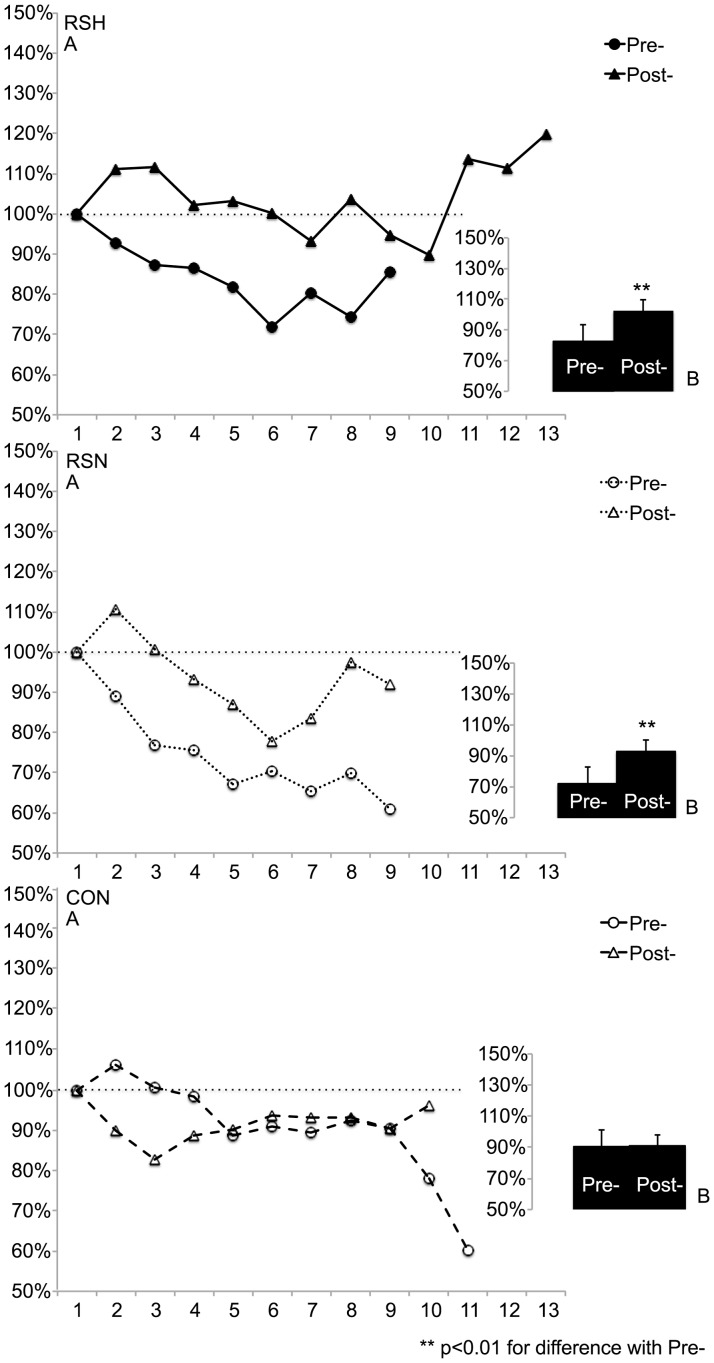
Δ[tHb]: successive changes in total hemoglobin concentrations’ amplitude during recovery phases between sprints (expressed in percent compared to the first recovery phase) measured by near infrared-spectroscopy (A) and Δ[tHb]_av_: average of all changes during the repeated sprint test to exhaustion (B) before (Pre-) and after (Post-) the specific repeated sprint training in hypoxia (RSH), in normoxia (RSN) or in control group (CON).

After training, Δ[tHb]_av_ increased to a similar extent in RSH and in RSN but was not different Pre- compared to Post- in CON (Fig. 5B).

Meanwhile, Δ[HHb]_av_ increased significantly only in RSH (from 98±7 to 115±12%, p<0.05) but not in RSN (105±12% vs. 116±10%, NS) nor in CON (109±8% vs. 113±13%, NS).

### Elextromyographic Activity

From Pre- to Post-, average RMS activity of the VL muscle was not different in RSH (0.53±0.23 vs. 0.50±0.16 mV, NS), in RSN (0.49±0.15 vs. 0.51±0.12 mV, NS) or in CON (0.53±0.21 vs. 0.51±0.19 mV). Similarly, from Pre- to Post-, average RMS activity of the BF muscle was not different in RSH (0.22±0.09 vs. 0.24±0.10 mV, NS), in RSN (0.20±0.08 vs. 0.22±0.09 mV, NS) or in CON (0.22±0.05 vs. 0.24±0.18 mV, NS). RMS activity of the VL across the RSA test decreased relatively to the first sprint (set to 100%) but to a similar extent in RSH (91±8% vs. 89±9%, NS), RSN (86±11% vs. 87±12%, NS) or in CON (89±10% vs. 88±6%, NS) in Pre- vs. Post-, respectively.

### mRNA Selected Gene Transcripts and Enzyme Activity

**Figure 6:**
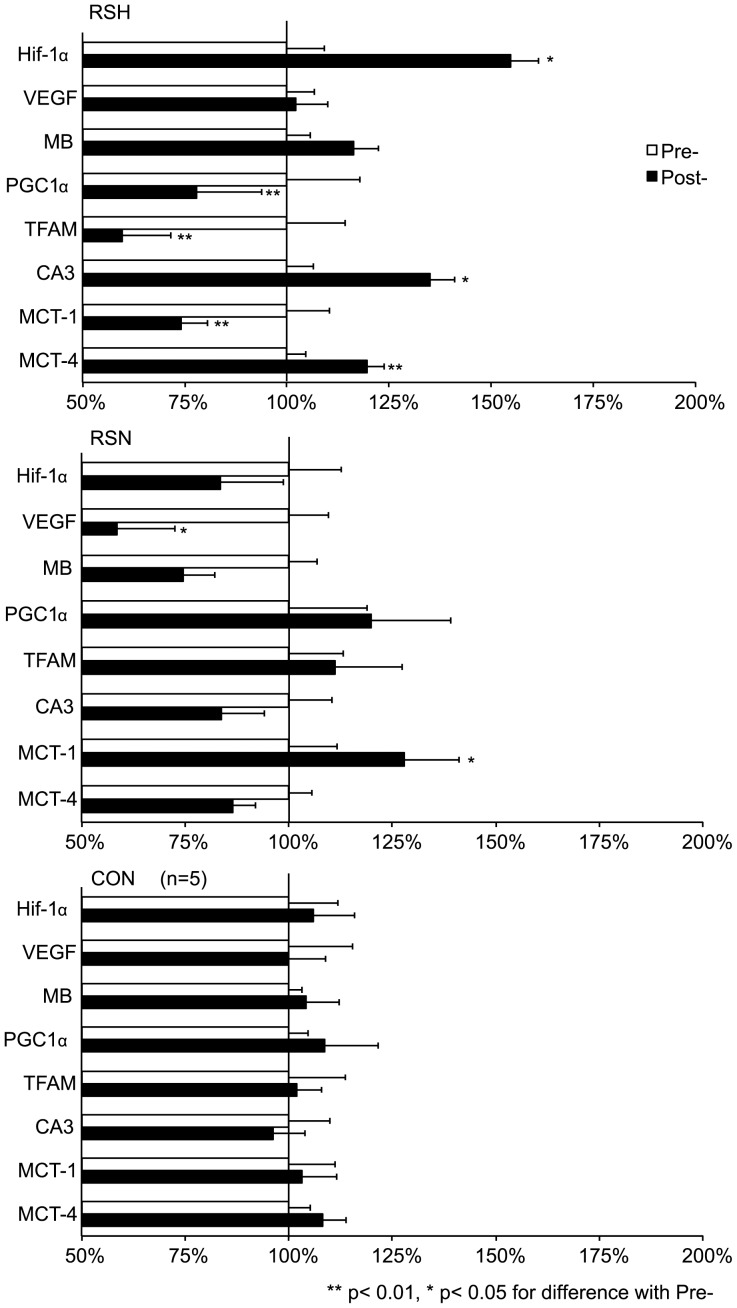
Relative mRNA expression of selected gene transcripts after 4 weeks of specific repeated sprint training before (Pre-) and after (Post-) the specific repeated sprint training in hypoxia (RSH), in normoxia (RSN) or in control group (CON). Open bars represent Pre- values and solid bars Post- values of mRNA concentrations in *vastus lateralis* muscle. Post- values were expressed in % compared to Pre- values (set to 100%). HIF-1α, hypoxia inducible factor-1α; VEGF, vascular endothelial growth factor; MB, myoglobin; PGC1-α, proliferator-activated receptor gamma coactivator-1α; TFAM, mitochondrial transcription factor A; CA3, carbonic anhydrase III; MCT-1, monocarboxylate transporter-1; MCT-4, monocarboxylate transporter-4; LDH, lactate dehydrogenase.

Figure 6 displays Pre- and Post- mRNA expression levels in RSH, RSN and CON groups. In Post- compared to Pre-, mRNA gene concentrations of hypoxia inducible factor (HIF-1α, +55%, p<0.05), carbonic anhydrase III (CA3, +35%, p<0.05), monocarboxylate transporter-4 (MCT-4, +20%, p<0.05) and lactate dehydrogenase (LDH, +12%, p<0.05) were augmented in RSH only. Conversely, mitochondrial transcription factor A (TFAM), peroxisome proliferator-activated receptor gamma coactivator 1α (PGC-1α) and monocarboxylate transporter-1 (−36%, p<0.01) concentrations were decreased in RSH only (−40% and −23% respectively, p<0.01) (Fig. 6). From Pre- to Post-, LDH activity was increased in RSH (63.6±13.8 vs. 71.3±14.2, p<0.05) but not in RSN (56.3±14.8 vs. 61.7±16.2, NS). Citrate synthase activity was not different and did not vary significantly in RSH (8.6±2.5 vs. 10.6±2.7, NS) and in RSN (8.5±3.1 vs. 8.5±3.8).

## Discussion

The principal novel finding of this investigation is that repeated sprint training in hypoxia allows further enhancement of repeated‐sprint performance to exhaustion than the same training in normoxia. Second, in RSH, the amplitude of blood flow variations during sprint phases was increased. Third, the increased mRNA expression of factors involved in pH regulation and glycolysis as well as the decrease in factors involved in mitochondrial biogenesis after RSH suggest a potential enhancement of the glycolytic (but not oxidative) activity in muscle.

### Repeated Sprint Ability and Cycling Performance Improvement

RSH and RSN elicited similar significant power improvements (∼6–7%) but hypoxic training only additionally delayed the decrease of power output and therefore extended the number of sprints performed. The ability to reach a higher PF and maintain it high during the last 5 s of the successive sprints explains the power improvements both in RSH and RSN. Indeed, maximal power production (e.g. during repeated sprints) is related to the ability to recruit more FT at very high PF [28] and FT are known to be essential in the power production when intensity increases [29]. Consequently, the observed increase in power can be attributed to an improved muscle recruitment strategy (e.g. additional FT motor units [30]) and inter-muscle coordination [22]. Noteworthy, like RSH, RSN improved average and maximal power output of the sprints; so the specific training performed is useful compared to CON in improving power during repeated sprints.

In addition, the aerobic performance (3-min all-out) was not modified after training (Table 4). Similarly, improvement of single 10-s sprint and Wingate performance did not differ between RSH and RSN. This was expected since intermittent training in hypoxia is known to be rather inefficient for additional performance improvements in endurance athletes, when compared to similar training performed in normoxia [6].

### Blood Flow Variations and Delayed Exhaustion during RSA Test

Δ[tHb] represents changes in blood volume in the muscle (i.e. blood perfusion) [31] so it is expected to vary widely during the RSA test. Since PF was higher in Post- and Δ[tHb] is influenced by cycle frequency [32], the increase in Δ[tHb]_av_ was expected and could contribute to higher power production both in RSH and RSN. Then, during the repeated sprint test, fatigue appeared rapidly, highlighted by the power decrement (∼33–35%, Fig. 3) throughout the set. At the muscular level, waste metabolites accumulation and energy supply are certainly essential limiting factors in RSA performance [33]. During RS phosphocreatine breakdown is very high [12] and inorganic phosphate (P_i_) accumulates in muscle. Since increased P_i_ levels may decline force, especially in FT recruited during such fatiguing exercise [34], an improved waste metabolites removal when blood flow is raised [35] might delay fatigue during a RSA test. This is in accordance with the greater increase in Δ[tHb]_av_ during sprints in RSH compared to RSN and the increase of the Δ[tHb] to power ratio, so it can be argued that blood perfusion was improved in RSH and could explain additional sprints performed in RSH.

Moreover, during sprinting, due to the high PF, FT are predominantly recruited [28], [36]. FT are mainly glycolytic [37] and contribute highly to the energy supply during RS [33]. More sprints in RSH could then be explained by an improved behavior of FT although it requires to optimize the contribution of anaerobic glycolysis known to be impaired as sprints are repeated [33]. Indeed, due to the poorer O_2_ delivery to working FT (compared to slow twitch (ST)) and their greater fractional O_2_ extraction if highly perfused [16], these fibers are likely to benefit more from the higher blood perfusion during sprints in RSH. This could help enhancing microvascular O_2_ delivery to FT, minimizing substrate level phosphorylation and intracellular perturbation (e.g. P_i_ accumulation and decreased pH), thereby “making FT to behave more like their oxidatively efficient ST counterparts” [38]. Then, with more FT recruited and working with less anaerobic energy dependence, fiber fatigue could potentially be diminished [39].

Interestingly, a recent study underlined a higher decrease in power output but with similar [tHb] kinetics during a RSA test performed in hypoxia compared to normoxia [40]. However, during our supervised training, RS power output did not decrease more in RSH than in RSN (since total work was similar (Table 3)). This could be explained by the training including only sets of 5 repeated sprints and therefore limiting power decrements. Then the improved [tHb] kinetics (i.e. Δ[tHb]) we observed might have been elicited by the greater exercise:rest ratio used (1∶2) inducing shortened recoveries.

When exercising, the O_2_ availability to demand ratio is lowered and muscle tissue oxygen delivery is maintained by an increased extraction of O_2_ in blood [41]. A more severe reduction of the ratio (e.g. during repeated sprints) requires an adapted arterial flow control (e.g. vasodilatation) to ensure adequate tissue perfusion [10]. In addition, during exercise in hypoxia the compensatory vasodilatation (with an increase in blood perfusion) aims at maintaining constant the total O_2_ delivery to the muscle [10]. Alongside, in hypoxia there is an increased sympathetic vasoconstrictor activity directed towards skeletal muscle [42] that occurs to a greater extent within FT (glycolytic type II fibers in rat) [43]. However, “the compensatory vasodilatation prevails over the vasoconstrictor response” and exercise intensity is essential in the amplitude of this compensatory vasodilatation [10]. So, maximal exercise intensities (e.g. in sprints) may maximize this amplitude. Though muscle fatigue attenuates the vasodilatory responsiveness [44] and the delayed fatigue during RSA test in RSH could then partly be due to an improved responsiveness of the vascular bed. Interestingly, blood flow and vascular conductance were shown to be augmented mostly in FT after dietary nitrate supplementation [43]. In accordance with the increase in Δ[tHb]_av_ in RSH (Fig. 4B), one may finally hypothesize that RSH enhancing predominantly FT behavior could participate to the improved blood perfusion through nitric oxide mediated vasodilatation mechanisms [10]. However, it must be admitted that data collected by NIRS might only represent muscle perfusion indirectly and should be interpreted with care. In addition, we did not observe significant differences in thigh muscle electromyography between groups. Nevertheless voluntary activation was reported not to differ during short-term hypoxia even at a higher altitude [45].

Still, the proposed mechanism based on the preferential recruitment and modified behavior of FT with an enhanced vasodilatory compensation induced in RSH is appealing as it could also explain why previous IHT studies [6]–[8] with lower exercise intensities failed to demonstrate additional benefits to endurance performance when compared to similar normoxic training. In these latter studies, FT fibers recruitment might not have been high enough since intensity was 2–4 fold lower than with the present RS training.

### Molecular Adaptations after Specific Training

Besides, performance and blood perfusion results are in accordance with the expression levels of some mRNAs. Our protocol including only brief periods of hypoxic training was sufficient for inducing a significant upregulation of HIF-1α. Such elevation in HIF-1α mRNA leads to a downstream activation of HIF-1 dependent pathway [46]. The subsequent increase in mRNA expression of CA3 and improved activity of LDH additionally suggests an increased capacity for pH regulation [47], although no significant difference in blood lactate was observed between Pre- and Post-. Indeed we also observed a decrease in mRNA expression of genes implicated in mitochondrial biogenesis such TFAM and PGC‐1α while LDH concentration was increased. Moreover, citrate synthase activity was not different between RSH and RSN suggesting that oxidative capacity was not different. Our study therefore indicates a shift from aerobic to anaerobic glycolytic activity in the muscle not in line with the previously suggested enhanced oxidative capacity after IHT [4], [5], [48]. In addition, we observed an upregulation of MCT-4 and a downregulation of MCT-1 in RSH. MCT-4 is mostly expressed in glycolytic tissues (i.e. FT) where it mediates lactic acid efflux [49] from the cells whereas MCT-1 is adapted to supply lactate to the cells for energy production [50]. Again, as MCT-4 (but not MCT-1) is upregulated by hypoxia [49], our results are in line with the hypothesis that more sprints performed during RSA were due mainly to modified FT fibers behavior. Although this study confirms an elevated HIF1-α mRNA expression after exercising at high-intensity during short exposures in hypoxia (and not in normoxia), the observed changes are largely different than the previously reported HIF-1 mediated muscle adaptations after IHT [5], [48] but underlines specific adaptations in RSH. Yet we also confirm the key role of exercise intensity *per se* during hypoxic training [9]. Overall, the upregulation of genes involved in oxygen signaling (HIF-1a), oxygen carrying (Mb) and pH regulation (CA3) and the concomitant downregulation of genes implicated in mitochondrial biogenesis (TFAM and PGC-1α) suggest a shift from aerobic to anaerobic glycolytic activity in the muscle.

### Conclusions

This study is the first to observe larger performance improvement after repeated sprint training in hypoxia than for to the same training in normoxia. Our main novel findings were that repeated sprint training in hypoxia leads to i) increased variations of blood perfusion possibly delaying fatigue during a RSA test and ii) specific molecular adaptations large enough for inducing further improvement in systemic RSA performance. Our results suggest an improved vascular conductance in repeated sprints to exhaustion where fast-twitch fibers are likely better utilized. Fatigue could potentially be delayed through FT working with less anaerobic energy dependence. In parallel to the increased blood perfusion and potentially better waste metabolites removal, modifications at the molecular level support a shift towards improved anaerobic glycolytic activity following RS training in hypoxia only.

In conclusion, this study reports the effectiveness of RSH for improving intermittent exercises performed in normoxia. The mechanisms are likely largely different than those previously associated to the inefficient IHT.
